# The effectiveness of health impact assessment in influencing decision-making in Australia and New Zealand 2005–2009

**DOI:** 10.1186/1471-2458-13-1188

**Published:** 2013-12-17

**Authors:** Fiona Haigh, Fran Baum, Andrew L Dannenberg, Mark F Harris, Ben Harris-Roxas, Helen Keleher, Lynn Kemp, Richard Morgan, Harrison NG Chok, Jeff Spickett, Elizabeth Harris

**Affiliations:** 1 Centre for Health Equity Training, Research and Evaluation, Part of the Centre for Primary Health Care and Equity, University of New South Wales, A Unit of Population Health, Sydney South West Local Health Disctricts, NSW Health, Sydney, New South Wales, Australia; 2 Southgate Institute for Health, Society & Equity, Flinders University, Adelaide, Australia; 3 University of Washington School of Public Health, Seattle, USA; 4 Centre for Primary Health Care and Equity, Faculty of Medicine, University of New South Wales, Kensington, Sydney, Australia; 5 Centre for Primary Health Care and Equity, University of New South Wales, Kensington, Australia; 6 School of Public Health and Preventive Medicine, Monash University, Melbourne, Australia; 7 Centre for Impact Assessment Research and Training (CIART), Department of Geography, University of Otago, Dunedin, New Zealand; 8 WHO Collaborating Centre in Environmental Health Impact Assessment and School of Public Health, Curtin University, Bentley, Australia

**Keywords:** Health impact assessment, Effectiveness, Evaluation

## Abstract

**Background:**

Health Impact Assessment (HIA) involves assessing how proposals may alter the determinants of health prior to implementation and recommends changes to enhance positive and mitigate negative impacts. HIAs growing use needs to be supported by a strong evidence base, both to validate the value of its application and to make its application more robust. We have carried out the first systematic empirical study of the influence of HIA on decision-making and implementation of proposals in Australia and New Zealand. This paper focuses on identifying whether and how HIAs changed decision-making and implementation and impacts that participants report following involvement in HIAs.

**Methods:**

We used a two-step process first surveying 55 HIAs followed by 11 in-depth case studies. Data gathering methods included questionnaires with follow-up interview, semi-structured interviews and document collation. We carried out deductive and inductive qualitative content analyses of interview transcripts and documents as well as simple descriptive statistics.

**Results:**

We found that most HIAs are effective in some way. HIAs are often directly effective in changing, influencing, broadening areas considered and in some cases having immediate impact on decisions. Even when HIAs are reported to have no direct effect on a decision they are often still effective in influencing decision-making processes and the stakeholders involved in them. HIA participants identify changes in relationships, improved understanding of the determinants of health and positive working relationships as major and sustainable impacts of their involvement.

**Conclusions:**

This study clearly demonstrates direct and indirect effectiveness of HIA influencing decision making in Australia and New Zealand. We recommend that public health leaders and policy makers should be confident in promoting the use of HIA and investing in building capacity to undertake high quality HIAs. New findings about the value HIA stakeholders put on indirect impacts such as learning and relationship building suggest HIA has a role both as a technical tool that makes predictions of potential impacts of a policy, program or project and as a mechanism for developing relationships with and influencing other sectors. Accordingly when evaluating the effectiveness of HIAs we need to look beyond the direct impacts on decisions.

## Background

Health Impact Assessment (HIA) is a tool used to assess impacts on social and environmental health determinants before embarking on proposed policies, plans, or projects [1-3]. HIAs, which may be undertaken at local, regional, national or international levels, are intended to inform decision-making. The use of HIA is supported by the World Health Organisation (WHO) which has called for assessment of the implications for health and the distribution of health impacts to be routinely considered in policy-making and practice [1,4-7].

Since the 1990s, there has been a rapid expansion in the use of HIA globally [8]. Many multilateral institutions now promote its use [8,9] and a plethora of guides exist [10]. Despite increasing use of HIA, its effectiveness is questioned. Case studies have demonstrated the effectiveness of HIA in influencing specific decisions [11-13]. The case studies also indicate that HIAs have a number of impacts beyond the specific decision.

There is a general and increasing recognition within both HIA and the broader field of impact assessment that viewing effectiveness in narrow terms, i.e. adoption and implementation of recommendations, overlook other important outcomes (e.g. raising awareness of health issues among decision-makers, establishing dialogue between stakeholders, and changing views and attitudes of stakeholders) and it misrepresents how decision-making works [14-19]. In addition, the complexity of effectiveness of HIAs has not been given due attention. To date four conceptual frameworks for evaluating HIA have been published in the literature. The first by Parry and Kemm [20] describes three domains to be examined in testing effectiveness: prediction, participation (involving stakeholders) and informing decision-makers. The framework has both process and outcome criteria. In practice there have been difficulties in evaluating the full range of potential outcomes and the extent to which benefits have been realised. The second framework by Birley suggests a fault analysis approach which focuses on identifying features that are responsible for the degree of success or failure of the HIA [21].

The third framework by Wismar et al. postulates four levels of effectiveness of HIA: direct effectiveness, general effectiveness, opportunistic effectiveness and no effectiveness [12] (see Table 1). Both the extent to which the HIA adequately addressed health impacts and the extent to which decisions have been modified as a result of the HIA, is considered. Although this framework is overly bureaucratic in focus it has strong face validity (estimate of the degree to which a measure is clearly and unambiguously tapping the construct it purports to assess) in recognising that a HIA may have more than one type of effectiveness, but also that the impact may not be linear and may not be directly attributable to the HIA. The Wismar framework has been used in two of the largest evaluations of the impact of HIA in decision-making [11,12]. It has also been used in the study described here.

**Table 1:** Four types of HIA effectiveness

		**Modification of pending decisions**	
		**Yes**	**No**
**Health adequately acknowledged**	**Yes**	*Direct effectiveness*	*General effectiveness*
		•HIA-related changes in the decision	•Reasons provided for not following HIA recommendations
		•Due to the HIA the project was dropped	•Health consequences are negligible or positive
		•Decision was postponed due to HIA	•HIA has raised awareness among policy-makers
	**No**	*Opportunistic effectiveness*	*No effectiveness*
		•The decision favouring health would have been made anyway	•The HIA was ignored
			•The HIA was dismissed

Source: [12].

**Figure 1:**
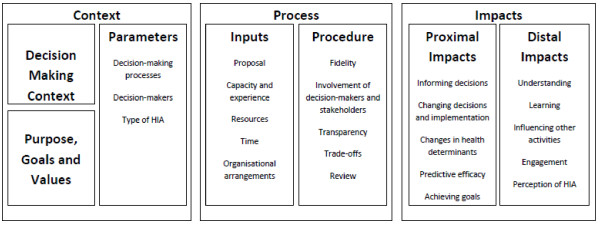
Conceptual framework for evaluating the effectiveness of HIA.

The fourth conceptual framework developed by two authors of this paper (BHR, EH) is based on a review of completed HIAs, literature and a local capacity building project [13] (see Figure 1). The framework will be familiar to evaluators because its structure conforms with a modified version of the Donabedian and Wisconsin evaluation frameworks [22]. It proposes three domains: context, process and impacts and reflects a growing recognition within the field that viewing effectiveness in narrow terms overlooks the distal, more indirect impacts and the process through which decisions are made. There is increased emphasis on the importance of learning as an impact of the HIA on participants’ understanding of health and how it is created, the development of specific skills that are transferable (such as assessing evidence) and the building of new relationships and partnerships.

We have carried out the first systematic empirical study of the influence of HIA on decision making and implementation of proposals in Australia and New Zealand. This paper focuses on two research questions:

1. Is there evidence that HIAs completed in Australia and New Zealand between 2005 and 2009 have influenced decision-making and the implementation of policies, program or projects to strengthen positive and mitigate negative health impacts?

2. What impacts do participants/stakeholders report following involvement in these health impact assessments?

Specifically it uses the Wismar framework to assess the effectiveness of 55 HIAs undertaken in Australia and New Zealand over this period and the Harris-Roxas and Harris framework to examine outcomes linked to effectiveness. We conclude by reflecting on the implications for HIA practice and draw out lessons on how HIA can be made more effective. Factors influencing the effectiveness of HIAs will be examined in a separate paper, this includes aspects of the survey data not included in assessments on decisionmaking.

## Methods

The research was led by an international team of 11 investigators from Australia (7), New Zealand (1), United States (2) and Thailand (1) supported by two researchers. Within the team there was a high level of expertise in HIA as well as experience with a wide range of methodologies and quantitative and qualitative methods

We used a two-step process first surveying 55 HIAs followed by 11 in-depth case studies. Multiple case study and survey methodologies were used for gathering and analysing qualitative and quantitative data [23]. The use of case studies aligns with our interest in capturing rich insights into the experiences and views of multiple stakeholders involved in HIA processes and enables examination of culture and context. Data gathering methods included questionnaires with follow-up interview, semi-structured interviews and document collation. We carried out deductive and inductive qualitative content analyses of interview transcripts and documents as well as simple descriptive statistics.

### Sampling

The process for identifying HIAs can be found in a previous publication [24] see Additional file 1. In this paper report on the results of a survey related to judgements of influence on decision-making and follow up interviews of these 55 HIAs (48 of 55 responded) and the 11 in-depth case studies see Additional file 2.

Information was collected from the HIA practitioners who had designed and implemented them, using a questionnaire and follow up interviews. The purpose of the questionnaire was to obtain information about the impact of the HIAs on decision-making as well as relevant contextual information not typically found in the HIA reports. These participants were purposively identified using authorship information provided in the reports. The 29 item questionnaire included a mix of open and closed questions that focused on their experiences and views with respect to four aspects of the HIAs they had undertaken: process, context, decision making and next steps see Additional file 3. Of a total of 55 HIAs we were able to complete the questionnaire for 48 participants (87%). We carried out 34 follow-up interviews which covered 42 of the HIAs. The interviews were carried out by either FH or HNC by telephone. The initial interviews (approximately six) were carried out by both researchers together, but the later interviews were conducted individually. Interviews were recorded, but not transcribed, with notes taken during the interviews.

To obtain an in-depth understanding of how effectiveness plays out within different contexts a purposive sub-sample of 11 case studies was selected using the criteria: (1) feasibility (access to stakeholders, willingness to participate); (2) representative of a range of effectiveness (assessed by investigators based on findings from first two phases of the study); and (3) mix of New Zealand and Australian case studies (see Additional file 2: HIAs included in study). Interviews were carried out with a minimum of three stakeholders from each HIA during 2011 (n = 33) see Additional file 4. We interviewed a mix of stakeholders from decision making organisations, those carrying out the HIA, and other key stakeholders such as steering group members. The interviews were semi-structured to ensure coverage of identified areas of interest but also allowing for new or emerging themes. A set of nine questions was used as the basis of the interview. Participants were asked to ‘tell the story of the HIA’ and were then prompted to talk in more detail (where necessary) about: the influence of the HIA; how successful the HIA was; factors affecting this; how stakeholders worked together; implementation of recommendations; timing; and learning. Interview duration varied from 25–90 minutes; most were approximately 45 minutes in length. The point at which data saturation (no new or relevant information or themes observed in the data) was reached was discussed at project meetings and work continued on the case and data analysed after saturation was reached.

Ethics approval was given by the UNSW Human Research Ethics Committee (23 April 2010). Written consent forms provided information about the project, purpose of the interview, conditions of consent including anonymity and contact details.

### Analysis

Descriptive analysis of closed questions was carried out using SPSS (SPSS Statistics 20). Factors were differentiated using the conceptual framework categories of context, process and outcomes [13]. HIAs were classified according to the Wismar effectiveness framework (direct, general, opportunistic, and no effectiveness, see Table 2) [12]. HIAs were assigned to a category based on our interpretation of the responses we received to the questionnaires and interviews. Where we were able to interview participants we asked them to categorise the HIAs or comment on our suggested categorisation. HIAs were categorized by the highest level of effectiveness they achieved.

**Table 2:** Wismar effectiveness categorisation with example quotes from questionnaire answers (N = 48)

**Direct effectiveness 31 (65%)**	**General effectiveness 11 (23%)**
A Community Education Project HIA *“directly affected the way the project was implemented, and the recommendations to address equity issues were incorporated in the project plan… The HIA ensured that vulnerable groups were identified and that health messages and activities were adjusted to reach and include these groups where possible*”.	*“The HIA was a component in a continuous loop of evidence-based learning practice that we sought to build internally and value externally in order to change traditional practice.... Gathering the evidence base was a powerful tool giving communities and councillors and staff a common understanding of the issues that required attention and an avenue to do this”.*
**Opportunistic effectiveness 3 (6%)**	**No effectiveness 3 (6%)**
*“I’m going to have to be honest here. … I still think it helped us. The process helped us put some things in we thought should have happened. We knew that. This was just a way of tacking them into the places with”.*	An HIA of a Health Promotion Plan was reported to have had no direct impact: “*I think the HIA was buried. Since that time an ex-staff member on the team of decision-makers has told me that it was rejected because it made people accountable to their decisions*!” However when asked whether they thought the decision would have been made without the HIA the same person responded negatively, saying that it “*made people think about equity implications more - which was the purpose of the HIA*”. They also felt the HIA had made a difference; it “*Made it clear that the planning process was flawed and inequitable”.*

HIAs which showed evidence of directly influencing the proposal, even when these changes were relatively minor, were categorised as directly effective. For example, an HIA that had at least one recommendation accepted and implemented was categorised as directly effective even if other recommendations were ignored. It should be noted that HIAs that were classified as directly effective may also have demonstrated general effectiveness, opportunism and, for some elements of the HIA, no effectiveness. HIAs classified as 'opportunistic' using the Wismar framework were particularly difficult to identify. This is partly due to a lack of clarity around what this category includes. The decision to assign a HIA to the ‘no effectiveness’ category was usually made because a participant reported that the HIA was not effective. During the categorisation process, we found that an individual HIA typically includes components that would fit into several categories, making it difficult to assign the overall HIA to a single category.

Case study interviews were recorded and professionally transcribed and then analysed using NVivo qualitative data analysis software (QSR International Pty Ltd. Version 9, 2010). The content analysis of interview transcripts was carried out using both predetermined categories as well as identification of emerging themes. Interviews were coded against the conceptual framework, effectiveness categories developed by Wismar (direct, general, opportunistic, and no effectiveness [25]), HIA stages, and emerging themes. FH initially coded and analysed data; 5 interviews were also coded by another investigator (EH or BHR) and compared to assess interrater reliability. Emerging results were written up and sent to the investigator team. EH then analysed specific themes (learning and timing) which had been challenged by other investigators as being unexpected or unclear. In addition EH reviewed FH’s coding for the conceptual framework and effectiveness categories. Coding tables for the conceptual framework were developed which contained themes with illustrative example quotes. These themes included the factors already included in the conceptual framework as well as themes that emerged from the data. In addition, some coding was carried out of HIA documentation including HIA reports, evaluations, papers, and other documents supplied by interviewees.

Data from the different methods were triangulated to develop an understanding of context, process and influence of the HIA as described in the Harris-Roxas, Harris conceptual framework (Figure 1) plus equity. The case studies summaries were then sent to a stakeholder from each HIA for comment and correction.

The research team carried out final analysis and evaluation of the research data. The project investigators and researchers met over two days to review findings of the study including coding and analysis of case study interviews; to develop a common understanding among investigators of key findings; and to identify limitations of the study. On the third day a validation workshop and webinar was carried out with investigators, HIA stakeholders, and jurisdictional representatives from Australia and New Zealand. Forty-seven people attended the workshop and thirty the Webinar. The workshop purpose was to test face validity of our analysis and discuss potential implications for policy and practice.

## Results

We have structured our results and discussion around the research questions.

### Is there evidence that HIAs completed in Australia and New Zealand between 2005 and 2009 have changed decision-making and the implementation of policies, program or projects to strengthen positive and mitigate negative health impacts?

**Figure 2:**
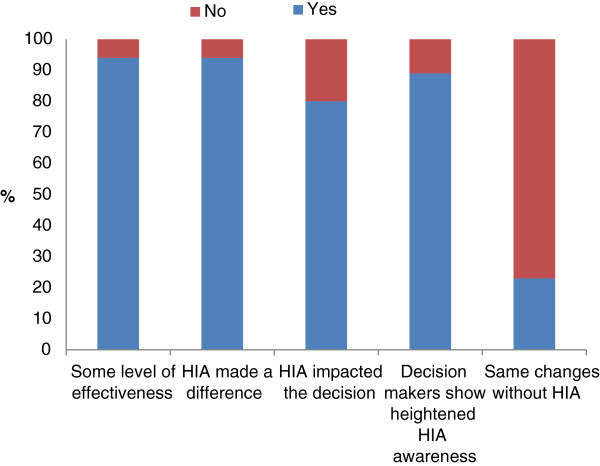
HIA effectiveness (percentage).

Almost all HIAs showed direct or general effectiveness (42, 89%) and 65% of HIAs were described as directly effective (31/47) (see Table 2). This was comparable to the US study that applied the Wismar framework [11]. Forty-four respondents (44/47, 94%) also believed that the HIA had made a difference. 80% (35/44,) of HIAs were reported to have impacted on the decision. Twenty-nine respondents (60% of all HIAs and 91% of those who responded) reported HIA-related changes being made to decisions. No one reported a decision being revoked or postponed because of the HIA. Of those who reported that the HIA did affect decision-making, 33 (33/40, 83%) reported that the HIA recommendations were easily incorporated into the planning process at the time. In 14 HIAs (14/36, 29%) it was reported that reasons were provided when recommendations were not followed. When asked to think about the changes that were made to decisions, just over 20% (10/43, 21%) reported that they were of the view that the same changes would have been made to the decision in the absence of the HIA (see Figure 2).

HIAs were reported to inform and in some cases lead to modification of the decision the HIA focussed on but also future decisions.

*“It’s what we set about to do - influence the statutory regional plan and that’s been done”.* (33)

*“Well it went to council. It was accepted by council. It was used to inform all the planning in [placename]* ”. (5)

“*HIA provided decision-makers with a structured way of thinking about unintended consequences and gave them confidence to make clear recommendations”.*

Often the HIAs were unable to influence whether or not a proposal went ahead but could influence the implementation of the proposal.

“So this study very much provided a fairly strong frame work in which to then develop contractual obligations under the project”. (23)

It was also reported that the HIAs can lead to expansions of decisions to include health considerations. There were also examples of important local health issues being identified through the HIA process.

*“we saw drafts on the way through as well to be able to comment on too so the HIA helped inform those as well*”. (1)

“*I have sighted the, the actual – I want to say the guts of the report – content of the report being recycled…I was gleeful when I saw that*”. (33)

“*a discovery that was looking at evidence in terms of cancer and the distance between waste dumps. This was something that – that sort of showed its head*”. (6)

### What impacts do participants/stakeholders report following involvement in these health impact assessments?

We found that HIAs affect participants in several different ways. Most respondents (40, 83%) reported that decision-makers showed heightened awareness of HIA after the process. This suggests that HIAs are having an effect on decision-makers as well as on decisions, which could result in longer-term influence and perhaps extend to future decisions.

A common outcome reported by participants is the learning that results from being involved in a HIA. That is they reported development of technical skills and knowledge (e.g. use of data/literature reviews, assessing evidence), conceptual learning (e.g. better understanding of the way their sector/work impacted on health, social determinants of health) and social learning (e.g. developing new relationships, learning about how to engage with other sectors, skills in negotiation). Learning was seen to occur through involvement in the HIA process but also through being a recipient of the HIA report, whereas relationship- and partnership-building were seen to occur through involvement in the HIA process. Again, this may influence the likelihood of other HIAs being conducted in the future. Furthermore the HIA process was seen to legitimise the involvement of the health sector in non-health sector decisions. It was also reported that the HIA process enables relationships between stakeholders to be formed or strengthened. In addition learning about HIA, health/ social determinants of health and the impact of their own sectors on health were reported.

Like I say, the more I learnt the angrier I got. HIA, if advocated right from the get go could save a lot of heartache, could save a lot of inconvenience and anger and anguish. It can do that. So I learnt that – I learnt – I certainly learnt a hell of a lot more in terms of urban development, et cetera, et cetera. In terms of my beloved suburb of [placename] I’ve learnt if it wasn’t for want of an HIA in its infancy we would not be in the situation, we would not be in catch-up mode right now. That’s what I learnt. (8)

Gathering the evidence base was a powerful tool giving communities and councillors and staff a common understanding of the issues that required attention and an avenue to do this”. (16)

“The HIA helped to share knowledge in the organisation between various stakeholders”. (31)

It succeeded in putting health on the agenda of a non-health organisation during the HIA process”. (47)

“the unintended outcome of the project was that it opened the whole conversation about how does Health comment on planning suggestions? It opened the conversation about where do we put Health? Do you leave it at the end as an assessment? Or do you put it at the front? So probably the unintended outcomes of the project might have, in fact, been –had a bigger impact than the actual project on the ground”. (36)

## Discussion

This study is significant because it goes beyond document analysis involving interviewing of (i) key decision-makers who were responsible for taking the recommendations forward and those who could influence them, (ii) the HIA assessors and (iii) other stakeholders involved in the process. A similar study involving interviews of HIA investigators, stakeholders and decision-makers is currently underway in the U.S. (http://www.rwjf.org/en/grants/grant-records/2011/04/identifying-the-critical-elements-of-successful-health-impact-as.html); investigators for this Australian study have shared information with the investigators of the U.S. study. This Australian study is also as far as we are aware the only attempt outside the US to conduct a census of all HIAs. This study provides an in-depth overview and analysis of the Australasian HIA context.

This research project has a number of limitations. Our sample is geographically specific. There may be important differences between the New Zealand and Australian context and other countries. We relied on participants’ perceptions, memories and own understanding of HIA effectiveness Because this was a new experience for many involved their perceptions may have been influenced by the novelty of the process.. There may be a tendency for less successful HIAs not to be reported or even completed. So although our sample showed a range of effectiveness it was biased towards ‘the winners’. We may have excluded HIAs that were done in-house, particularly by the private sector for internal license to operate requirements as opposed to regulatory ones. Our sample was limited to 55 HIAs in phase one and then down to 11 case studies. Two of the 11 case studies were incomplete (The Christchurch case study only had one key informant interview due to scheduling problems, The Goodooga Case Study did not proceed due to timing problems as the community was isolated for several weeks by flood). We interviewed on average three people from each case study. Perhaps more data could strengthen our findings; however, we did collect a significant amount of data and reached a point of data saturation in our analysis.

Study participants reported multiple perspectives on what effectiveness means in the context of HIA, the effectiveness of specific HIAs, and the salience of different factors in influencing effectiveness. We found that most of the HIAs were judged to be effective in some way using the Wismar framework. They also led to other changes (general effectiveness) such as informing follow-on or related decisions and raising awareness among decision-makers of health impacts, determinants of health and the affect health of their sector. Direct and indirect impacts are outlined in Table 3.

**Table 3:** Reported outcomes of HIA

**Direct/proximal**	**Indirect/distal**
**Inform**	**Technical learning**
Decision making	Literature reviews
Future decisions	Use of data
Implementation	Assessment of evidence
Adapted	Capacity building
**Modify**	**Conceptual learning**
Decisions	Social determinants of health
Related decisions	Relationship between their area and health
Follow on decisions	Perceptions of usefulness of HIA
Implementation	Use of evidence
	Awareness (decision makers, wider community)
**Expand decision making**	**Social learning**
Inclusion of health/ determinants of health	Partnerships/relationships
	Decision making processes
	Legitimacy

The learning reported by decision-makers suggests that HIAs are having an effect on decision-makers as well as on decisions, which could result in longer-term influence and potentially extend to future decisions. Most of the HIAs in the study were decision support which implies there is a close relationship with decision makers that may mean that they are more likely adopt recommendations and experience learning as an outcome of the process. It was also reported that the HIA process enables relationships between stakeholders to be formed or strengthened. Again, this may influence the likelihood of other HIAs being conducted in the future. A finding of the study is that learning is an important outcome for HIA participants but is poorly articulated as valued impacts of HIA.

We found it difficult to assign the HIAs to the Wismar effectiveness categories. We found that our HIAs fit in multiple categories, with different aspects of HIAs achieving different levels and types of effectiveness. Attempting to assign categories on the basis of ‘yes’ or ‘no’ led to somewhat arbitrary decisions that did not always reflect our overall understanding of the effectiveness of the project. The conceptual framework by Harris-Roxas and Harris (see Figure 1) was able to capture the complexity of the assessment process although there would need to be a discussion by the Steering Group at the screening stage on the meaning of various terms, such as trade-offs, to facilitate common understandings. The articulation of direct and indirect impacts and types of learning providing a clear framework for analysis. However as the framework was developed in response to perceived limitations in the detail for assessment in the Wismar framework it has identified too many categories to be routinely assessed and now needs to be simplified to include fewer and more critical elements for success. In addition there was insufficient focus on the role of the individuals in facilitating the effectiveness of HIA.

We were able to expand our understanding of the dimensions of direct effectiveness to include influencing as well as changing decisions, broadening the range of impacts that were considered and directly impact on the social determinants of health. The use of the conceptual framework to analyse the case studies allowed us to better identify the range of indirect impacts of the HIA which could broadly be seen as technical, conceptual and social learning. These were also the factors identified by participants as the impact of their involvement in the HIA.

## Conclusions

The study has clearly demonstrated the direct and indirect effectiveness of available HIA in Australia and New Zealand. It suggests that public health leaders and policy makers should be promoting the use of HIA and investing in building capacity to undertake high quality HIAs.

HIAs are often directly effective in changing, influencing, broadening areas considered and in some cases having an immediate impact on outcomes. Even when HIAs are reported to have no direct effect on a decision they are often still effective in influencing decision-making processes. But participants saw effectiveness as much broader than direct impacts on decisions. Many saw changes in relationships, better understanding of the determinants of health and positive working relationships as major and sustainable impacts of their involvement.

This finding raises an important issue in relation to seeing HIA as a technical tool that makes predictions of potential impacts of a policy, program or project or as a mechanism for developing relationships with and influencing other sectors. Focusing on indirect impacts such as relationship building at the expense of neglecting the systematic analysis and prediction of impacts to influence decision making runs the risk of ignoring some of the essential elements of HIA; assessing health and equity impacts, structured stepwise process, making recommendations [26]. Conversely focussing solely on the technical aspects may risk ignoring potential areas such as relationships and learning which may have even greater long term impact.

Our study also found that effectiveness is not a static concept. Goals can change during the process and be refined to reflect what it is possible to influence over time. Different stakeholders can hold contradictory views on the effectiveness of a HIA. A HIA may be effective in terms of achieving one stakeholder’s goals but not another’s. Judging effectiveness in the achievement of intended outcomes is potentially problematic in HIA. This also speaks of the need for longitudinal studies of the effectiveness of HIA so that any changes to their original purposes, and the factors underlying them, can be tracked and analysed. Finally, there is still a need for more research to understand the factors associated with enhanced or diminished effectiveness.

## Competing interests

The authors declare that they have no competing interests.

## Authors’ contribution

EH, FB, BH-R, LK, JS, HK, RM, MH, AMW, ALD are investigators on the ARC discovery grant funded project that this paper originates from. All of the investigators were involved in conceptualizing and designing the project. Fiona Haigh and Harrison Ng Chok are researchers employed on the project. All of the authors reviewed at least one of the 55 HIA reports and the 11 case studies that form the basis for this article (data analysis). HNC reviewed all of the HIA reports. FH collected the data for the case studies and carried out preliminary analysis of the survey and case study data. All of the authors significantly contributed to the analysis and interpretation of the data. Progress reports were circulated to the project group and discussed at regular project meetings. In June 2012 all of the project team met for two days to analyze and interpret the project findings. This included reviewing the reviews and in depth discussion covering interpretation, quality of and utility of the review package, project methodology (strengths and limitations), key findings and implications for policy, practice and research. In addition an early draft of the paper was reviewed and discussed by the whole project group at this meeting. FH and EH took a lead on drafting and revising the article. All of the authors were involved in conceptualizing the article (during discussions at the regular project meetings). Since then the article has been circulated to the whole project group for comment and then revised and re-circulated for further comment. All of the project team has critically revised and made detailed comments on multiple versions of the article. All authors read and approved the final manuscript.

## Pre-publication history

The pre-publication history for this paper can be accessed here:

http://www.biomedcentral.com/1471-2458/13/1188/prepub

## Supplementary Material

Additional file 1 Phase 1 inclusion diagram. Click here for file

Additional file 2 HIAs included in study (case study HIAs = bold). Click here for file

Additional file 3 Questionnaire. Click here for file

Additional file 4 Case study interview questions. Click here for file
