# Supplementary figures and images for: The effectiveness of health impact assessment in influencing decision-making in Australia and New Zealand 2005–2009
Source: BMC Public Health. 2013 Dec 17;13:1188. doi: 10.1186/1471-2458-13-1188 (PMC3878483; doi:10.1186/1471-2458-13-1188)

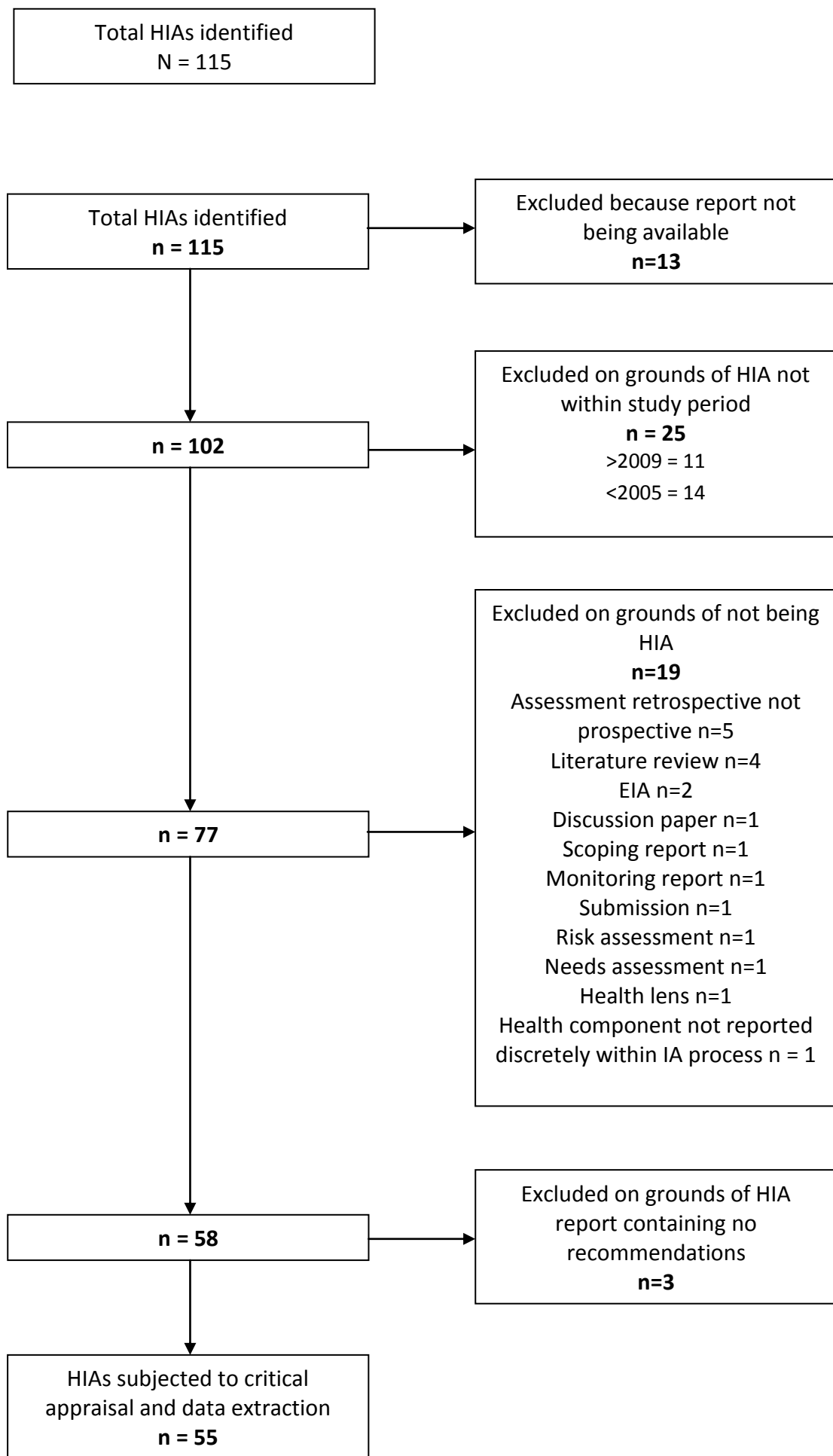

Supplement: Additional file 1 — Phase 1 inclusion diagram. [file 1471-2458-13-1188-S1.pdf]
