# Supplementary material for: The effectiveness of health impact assessment in influencing decision-making in Australia and New Zealand 2005–2009
Source: BMC Public Health. 2013 Dec 17;13:1188. doi: 10.1186/1471-2458-13-1188 (PMC3878483; doi:10.1186/1471-2458-13-1188)
Supplement: Additional file 2 — HIAs included in study (case study HIAs = bold). [file 1471-2458-13-1188-S2.pdf]

Table 1 HIAs included in study (bold = case study HIAs)

Year	State/Region	Name of HIA	Description	Part of capacity-building project	Policy, Plan, Program, Project	Depth	Sector
**2009**	**QLD**	**EFHIA of alternative patterns of development of the Whitsunday Hinterland and Mackay Regional Report**	**Area plan to influence land-use planning**	**No**	**Plan: options**	**Rapid**	**Land-use**
**2009**	**NSW**	**EFHIA of the review of Goodooga Health Service**	**Plan to change the an implementation plan to change a rural health service**	**No**	**Plan: options**	**Intermediate**	**Health service**
2009	Hawke’s Bay	Whakawateatia Hawke's Bay District Health Board: HIA on the proposed Air Quality Plan Change	Plan to change air quality standards	Yes	Plan: options	Rapid	Air
2009	Wellington	Wairarapa Non-fluoridation of water WOHIA	Plan to implement a water fluoridation plant to supply two local governments	Yes	Plan: options	Desktop	Water
2009	Wellington	HIA of Makoura Responsibility Model	Project aimed to encourage self-responsibility and self-management in students to deal with challenging behaviour.	Yes	Project	Rapid	Education
2009	Hawke’s Bay	HIA on the draft Wairoa District Council Waste Management Activity Management Plan	Plan to implement a waste management plan	Yes	Plan: options	Desktop	Waste Manage-ment

**2009**	**Hawke's Bay**	**HIA on Flaxmere Town Centre Urban Design Framework Proposal**	**Area plan to influence land-use planning**	**Yes**	**Plan: options**	**Desktop**	**Land-use**
**2009**	**Hawke's Bay**	**HIA on implementation of Oral Health Strategy: Location of a community clinic in Flaxmere**	**Project aimed to implement oral health strategy**	**Yes**	**Project**	**Intermediate**	**Health service**
2009	Waikato	Age-Friendly Community Shaping the Future for Waihi beach	Plan to inform the future land-use of a beachside community	Yes	Plan: options	Intermediate	Community Service
**2009**	**Auckland**	**Regional Land and Transport Strategy HIA**	**Project to implement a regional land transport strategy**	**No**	**Policy**	**Comprehensive**	**Transport-ation**
2009	Auckland	Manukau Built Form and Spatial Structure Plan HIA Report	Land-use plan for a particular area	Yes	Plan: options	Intermediate	Land use
2008	SA	EFHIA South Australian Better Health Initiative School and Community Initiatives	Implementation of a state-wide school and community initiative	No	Program	Intermediate	Health service
2008	QLD	HIA Flinders Street Redevelopment Project	Program to look at redevelopment of a CBD main street	No	Project	Rapid	Land-use
2008	NSW	Good for Kids, Good for Life EFHIA	Implementation of a state-wide school and community initiative	Yes	Program	Rapid	Health service
**2008**	**NSW**	**HIA Lithgow City Council Strategic Plan**	**Plan to implement land-use Strategic plan from local government**	**No**	**Plan: options**	**Intermediate**	**Land-use**
**2008**	**VIC**	**Leopold Strategic Footpath Network HIA**	**Plan to implement a strategic footpath network in CBD**	**No**	**Project**	**Intermediate**	**Land-use**
2008	NSW	Oran Park and Turner Road	Land-use plan aimed at a	No	Plan: options	Intermediate	Land-use

		HIA	particular area				
2008	VIC	A Matter of Equity — Case Study, Frankston City Council	Land-use plan aimed at a particular area	No	Program	Rapid	Food
2008	VIC	SHIA of Dandenong High School Doveton Campus closure	Plan to for a school closure and amalgamation of its students into other schools in the area	No	Project	Intermediate	Education
2008	NSW	SIA Potts Hill	Plan to redevelop a particular area	Yes	Plan: options	Rapid	Land-use
2008	WA	Health Impacts of Climate Change Adaptation Strategies for WA	Plan of scenarios and state-wide strategies to adapt to climate change	No	Plan: options	Intermediate	Climate change
2008	Canterbury	HIA Central Plains Water Scheme	Proposed irrigation scheme for 60,000 ha of land	Yes	Plan: options	Rapid	Water
2008	Wellington	HIA of Regional Policy Statement Regional Form and Energy Draft Provisions	Policy implemented on regional land-use and energy provision	No	Policy	Rapid	Land use
2008	Auckland	Ranui Urban Concept Plan HIA	Land-use plan for a particular area	No	Plan: options	Intermediate	Land use
2008	Auckland	HIA McLennan Housing Development	Plan to implement housing development types	No	Plan: options	Rapid	Housing
2008	Otago	Proposed Liquor Restriction Extensions in North Dunedin HIA	Plan to implement liquor restrictions in student neighbourhood	Yes	Policy	Intermediate	Harm minimis-ation
2008	Waikato	Tokoroa Warm Homes Clean Air Project: Health and Well-being Impact Assessment	Plan to change air quality standards	No	Plan: options	Rapid	Air
2008	Hawke's Bay	HIA on Draft Hastings district Council Graffiti Vandalism Strategy	Program to implement graffiti strategies	Yes	Plan: options	Rapid	Community Service
2007	VIC	HIA Hobson Bay Urban Greywater Diversion Project	Land-use plan for a particular area	No	Plan: options	Rapid	Water

2007	NSW	HIA Coffs Harbour Our Living City Settlement Strategy	Land-use plan for a particular area	No	Plan: options	Intermediate	Land-use
2007	NSW	Greater Western Sydney Urban Development HIA	Land-use plan for a particular area	Yes	Policy	Intermediate	Land-use
2007	NSW	HIA of Redevelopment of Liverpool Hospital	Project to redevelop a hospital	Yes	Project	Intermediate	Health service
2007	WA	HIA of Landfill Site and Housing Development in Mundijong, WA	Plan to introduce a landfill site and housing development	No	Plan: options	Intermediate	Land-use
2007	NSW	HIA of Car Park Waste Encapsulation Remediation	Plan to implement waste remediation for a car park	Yes	Plan: options	Rapid	Waste Manage-ment
2007	QLD	SIA of Gatton Correctional Facility	Plan to implement a Correctional facility	Yes	Plan: options	Comprehensive	Institution
2007	NSW	HIA on Rural Health Service Redesign Proposal	Plan to redesign a rural health service	Yes	Plan: options	Comprehensive	Health service
**2007**	**NSW**	**Bonnyrigg Living Communities SIA**	**Land-use and regeneration for a particular area**	**No**	**Project**	**Comprehensive**	**Housing**
2007	Far North	Kerikeri-Waipapa Draft Structure Plan	Land-use plan for a particular area	No	Plan: options	Intermediate	Land-use
**2006**	**NSW**	**HIA of Greater Granville Regeneration Strategy**	**Land-use and regeneration for a particular area**	**Yes**	**Plan: options**	**Rapid**	**Land-use**
2006	NSW	HIA of indigenous environmental health workers	Plan to introduce an indigenous environmental health worker	Yes	Plan: options	Intermediate	Health service
2006	NSW	Rapid Equity Focused HIA of the Australian Better Health Initiative: assessing the NSW components of priorities 1 and 3	Health program to be implemented across a state	No	Program	Intermediate	Health service
2006	NSW	Wollongong Foreshore Precinct Plan	Land-use plan for a particular area	Yes	Plan: options	Desktop	Land-use

**2006**	**NSW**	**Bungendore HIA: urban development in a rural setting**	**Land-use plan for a particular area**	**Yes**	**Plan: options**	**Rapid**	**Land-use**
2006	NSW	SIA of Lower Hunter Regional Strategy	Land-use plan for a regional area	Yes	Plan: options	Intermediate	Land-use
2006	VIC	HIA in the East Gippsland Shire Council five-year arts and culture strategic plan	Art and cultural program implemented for local government	Yes	Program	Desktop	Community Service
2006	VIC	HIA in the East Gippsland Shire Council Kerbside Waste Collection Strategy	Plan to implement a waste management plan	Yes	Program	Rapid	Waste Manage-ment
2006	NSW	HIA on an integrated disease prevention campaign	Plan to implement an integrated disease prevention campaign	Yes	Plan: options	Rapid	Health service
2006	Marlborou gh	Wairau Road Widening HIA	Project to expand a suburban road works	No	Project	Rapid	Transport-ation
2006	Wellington	HIA of Greater Wellington Regional Land Transport Strategy	Policy implemented on regional land use	No	Policy	Rapid	Transport-ation
2006	New Zealand	HIA Healthy Wealthy and Wise Future Currents Electricity Scenarios 2005-2050	Plan to implement new energy saving provisions	No	Plan: options	Intermediate	Energy
**2006**	**Canterbury**	**HIA Greater Christchurch Urban Development Strategy options**	**Land-use plan aimed for a particular area**	**No**	**Plan: options**	**Rapid**	**Land-use**
2006	Marlborou gh	SIA of the Draft Nelson City Council Gambling Policy	Implementation of a gambling policy	No	Policy	Rapid	Harm minimis-ation
2006	Auckland	HIA of Mangere Growth Centre Plan	Plan to manage growth	No	Plan: options	Rapid	Land-use
2005	QLD	HSIA of South East Queensland Regional Plan	Prospective population state-wide strategies	No	Plan: options	Comprehensive	Land-use
2005	Auckland	Avondale's Future Framework	Land-use plan for a particular	Yes	Plan: options	Rapid	Land-use

Rapid HIA	area
