# Supplementary material for: The effectiveness of health impact assessment in influencing decision-making in Australia and New Zealand 2005–2009
Source: BMC Public Health. 2013 Dec 17;13:1188. doi: 10.1186/1471-2458-13-1188 (PMC3878483; doi:10.1186/1471-2458-13-1188)
Supplement: Additional file 3 — Questionnaire. [file 1471-2458-13-1188-S3.pdf]

The Effectiveness of Health Impact Assessments Conducted in Australia and New Zealand

Thank you for agreeing to participate in our study. We are interested in your experiences in being involved in the HIA and your views on what factors have impacted on the take up and implementation of the HIA's recommendations.

Please think about the HIA that you have been asked to talk about. Make a note of any questions that are not clear or you find difficult to answer and we can discuss them at the follow up phone call. Before starting please read the information sheet and complete the consent form.

You can either fill it in online by typing in your answers and putting an 'x' next to appropriate boxes and emailing to f.haigh@unsw.edu.au or, alternatively, print out and hand write your answers and either fax (+61 2 9612 0762) or scan and email.

If you have any questions please contact Fiona Haigh (phone +61 2 9612 0779, fax +61 2 9612 0762, email f.haigh@unsw.edu.au).

HIA Process

Q1 Can you please briefly describe why the HIA was undertaken?

____

____

____

____

____

Q2 Can you please briefly describe your role in the HIA?

____

____

____

____

____

Q3 Before this HIA, did you have any previous HIA experience?

☐ *Yes* ☐ *No*

If yes- please briefly describe

____

____

____

Q4 Has the decision the HIA was intended to influence been made yet?

☐ *Yes* ☐ *No*

Q5 At what point in the decision making process for the Policy, Plan or Project was the HIA completed?

☐ *Completed prior to decision making process*

☐ *Completed during decision making*

☐ *Completed after decision making*

☐ *Not intended to be timely*

Q6 Was there community involvement in the HIA?

☐ *Yes* ☐ *No*

If YES please tick applicable answers

☐ *Community involvement steering group*

☐ *Community involved in doing assessment*

☐ *Community involvement commissioning*

☐ *Community involvement in providing primary data (interviews, focus group etc)*

☐ *Community involvement in prioritizing impacts*

☐ *Community involvement developing recommendation*

☐ *Community involvement as decision makers*

Q7 How were findings presented to community?

- ☐ *not reported*
- ☐ *written report only*
- ☐ *written report plus presentation*
- ☐ *presentation only*
- ☐ *Summary report*
- ☐ *summary report and presentation*
- ☐ *Other*

____

____

Q8 How were findings presented to decision makers?

- ☐ *not reported*
- ☐ *written report only*
- ☐ *written report plus presentation*
- ☐ *presentation only*
- ☐ *summary report*
- ☐ *summary report and presentation*
- ☐ *Other*

____

____

Q9 Did the decision maker review the report?

Yes	*No*	*Still being reviewed*
❑	❑	❑

Q10 Did the decision makers provide information about their decisions in relation to HIA recommendations?

- ☐ *Yes*
- ☐ *No*

If YES in what format?

- ☐ *Report*
- ☐ *Letter/email*
- ☐ *Verbal*
- ☐ *other*

____

____

Q11 Has process and/or impact evaluation of the HIA been planned or carried out?

**impact evaluation**	**process evaluation**
☐ planned	☐ planned
☐ completed	☐ completed

If yes- please briefly describe main evaluation findings

___

___

___

Is there a copy of the evaluation report available?

☐ *Yes* ☐ *No*

Q12 Has monitoring of the HIA recommendations been carried out?

Yes	*No*	*Ongoing*
❑	❑	❑

HIA Context

Q13 Was there any controversy and/or opposition at the time of the HIA to the Policy, Plan or Project?

☐ *Yes* ☐ *No*

If YES would you say the level of controversy/opposition was?

	low	*Some*	*Medium*	*High*
controversy	❑	❑	❑	❑
opposition	❑	❑	❑	❑

Q14 Did the decision makers support the HIA process?

☐ *Yes* ☐ *No*

If yes - please briefly describe how

____

____

____

Q15 Did the decision makers have prior HIA experience?

☐ *Yes* ☐ *No*

Q16 Were there other groups/ stakeholders making the same or similar recommendations as the HIA?

☐ *Yes* ☐ *No*

If yes - please identify groups/stakeholders

____

____

____

Q17 Were the health impacts identified

Generally positive	*Generally negative*	*Evenly split*
❑	❑	❑

Q18 What were the types of health impacts identified

Physical health	*Mental or psychological*	*Social health*
❑	❑	❑

Q19 On a scale of 1 to 5 how would you rate the health impacts identified?

1	*2*	*3*	*4*	*5*
Insignificant	*Minor*	*Moderate*	*Severe*	*Very severe*
❑	❑	❑	❑	❑

Q20 Was an Environmental Impact Assessment carried out?

☐ *Yes* ☐ *No*

HIA decision making

Q21 Did the HIA impact on the actual decision?

☐ *Yes* ☐ *No*

If YES – in what way?

☐ *HIA related changes in decision (e.g. recommendations taken on board)*

☐ *Due to HIA the project was stopped*

☐ *Decision postponed due to HIA*

☐ *Decision supported by HIA*

☐ *Other – please elaborate*

Comments

____

____

____

Q22 If YES - were the recommendations easily incorporated into the planning process at the time?

☐ *Yes* ☐ *No*

Q23 If NO - Why do you think the HIA did not have an impact on the decision?

____

____

____

Q24 Were reasons given for HIA recommendations that were not followed?

☐ *Yes* ☐ *No*

If YES – What were the reasons given?

____

____

____

Q25 Thinking now about changes to the decision. In your view, without the HIA, would the same changes to the decision have been made?

☐ *Yes* ☐ *No*

Why?

____

____

____

Q26 Was there evidence of heightened HIA awareness in decision makers? (i.e. Raised awareness in decision maker of relationship between health and determinants of health, increasing likelihood of consideration of health consequences in deliberations)

☐ *Yes* ☐ *No*

Please describe

____

____

____

Q27 In your view, did the HIA make a difference?

☐ *Yes* ☐ *No*

Why- in what way?

____

____

____

____

____

____

____

HIA next steps

Q28 For the next phase of the project we are going to carry out up to 12 case studies of HIAs in order to gain a more indepth understanding of the factors that influence the effectiveness of HIA. Would you be willing to be involved in a case study?

☐ *Yes* ☐ *no*

Could you provide us with contact details of other stakeholders (e.g. steering group members)?

____

____

____

Q29 Do you have any other comments about the effectiveness of the HIA?

____

____

____

____

____
