# Supplementary material for: The effectiveness of health impact assessment in influencing decision-making in Australia and New Zealand 2005–2009
Source: BMC Public Health. 2013 Dec 17;13:1188. doi: 10.1186/1471-2458-13-1188 (PMC3878483; doi:10.1186/1471-2458-13-1188)
Supplement: Additional file 4 — Case study interview questions. [file 1471-2458-13-1188-S4.pdf]

Interview questions: Understanding the impact and effectiveness of Health Impact Assessment

Background

The Centre for Health Equity Training, Research and Evaluation (CHETRE) at the University of New South Wales has been funded by the Australian Research Council to study the effectiveness of Health Impact Assessments (HIA) conducted in Australia and New Zealand. The study's aim is to describe and explain changes to decision-making and implementation associated with the use of HIA in Australia and New Zealand between 2005 and 2009. In phase one of the study we reviewed all the identified HIAs and carried out a survey and interviews to identify factors influencing the effectiveness of HIA. In phase 2 (this phase) twelve Health impact Assessments have been selected for a more in depth case study. We are exploring different stakeholders' perspectives on HIA effectiveness and testing theories about HIA effectiveness that we have developed.

Format

The interview will be semi-structured using questions as a starting point for a conversation where we can learn from each other (and I can test ideas with you). I am more than happy to discuss issues as they arise, including any contradictory issues you may have. What effectiveness in HIA means and what factors influence this can be difficult to pin down conceptually, so examples would be welcome where possible.

The interview is designed to last no more than an hour, although we can speak for more than this if you feel this is necessary and you have the time.

Questions

1. How was HIA undertaken? What happened?
2. What changed as a result of doing the HIA?
3. What was the purpose and expected outcomes of the HIA?
4. Was the HIA a success? Why?
5. In general, can you please describe what would make a HIA successful?
6. Can you please tell me more about how the different stakeholders worked together
    a. What changed as a result of this?
7. Can you please tell me more about who was responsible for implementing the HIA recommendations and how they were involved in the HIA
8. Can you please tell me more about the timing of the HIA?
    a. What was the (broader) context within which the HIA took place and how did this influence the decision to carry out the HIA and the HIA process itself?
9. What did you learn from doing the HIA?
